# Telocytes in inflammatory bowel diseases: contributions to pathology and therapeutic potentials

**DOI:** 10.3389/fcell.2024.1452258

**Published:** 2025-01-13

**Authors:** Ronaldo Paolo Panganiban, Christina McAninch, Marina Chulkina, Irina V. Pinchuk

**Affiliations:** Division of Gastroenterology and Hepatology, Department of Medicine, Penn State College of Medicine, Hershey, PA, United States

**Keywords:** telocyte, fibrosis, IBD, ulcerative colitis, Crohn’s disease

## Abstract

Telocytes, a novel mesenchymal cell population, are characterized by their distinctive long and slender projections known as telopodes and have garnered significant interest since their formal introduction to the literature in 2010. These cells have been identified in various tissues, including the gastrointestinal (GI) tract, where they are suggested to play important roles in maintaining structural integrity, immune modulation, and barrier function. Inflammatory bowel diseases (IBD), which include Crohn’s disease (CD) and ulcerative colitis (UC), are characterized by chronic inflammation and fibrosis. While limited information is available on the fate of telocytes in this group of diseases, it has been suggested that loss/plasticity of telocytes can be among the key factors contributing to their pathogenesis. This review focuses on the current understanding of telocytes, their structural features, and their distribution within the GI tract under gut homeostasis and IBD. We also discuss the roles of these cells in immune regulation and intestinal repair. We highlight evidence implicating telocytes in the pathogenesis of IBD and other chronic inflammatory diseases that share similar pathophysiological processes with IBD. Lastly, we discuss the current challenges in gut telocyte biology and the potential therapeutic implications of telocytes in IBD.

## 1 Introduction

Telocytes, a previously unappreciated population of mesenchymal cells characterized by their distinctive long and slender projections known as telopodes, were formally introduced to the scientific literature by Dr. Popescu and Dr. Faussone-Pellegrini in 2010 (Popescu and Faussone-Pellegrini, 2010a). Previously, these cells were referred to as “interstitial Cajal-like cells” due to their morphological similarities to the interstitial cells of Cajal, which are known for their role in gut motility. Since the seminal publication by Popescu and Faussone-Pellegrini, the interest in telocytes has grown steadily over the last decade, resulting in 643 Pubmed-indexed publications at the time of this writing.

Telocytes have been identified in several tissues, including the central and peripheral nervous systems, heart, respiratory tract, urinary tract, and GI tract, among others (Varga et al., 2019). Changes in telocyte number and phenotype have been observed in chronic inflammatory (Ibba-Manneschi et al., 2016) and fibrotic diseases (Wei et al., 2022) as well as during neoplastic transformation (Aleksandrovych and Gil, 2021). More recently, telocytes have been suggested to play a role in the pathogenesis of IBD.

IBD, which includes Crohn’s disease (CD) and ulcerative colitis (UC), is a relapsing and remitting group of multi-factorial chronic inflammatory diseases with increasing prevalence worldwide (Wang et al., 2023). Although the exact etiology of IBD remains unclear, over the years, it has become apparent that genetic predisposition, environmental exposure, alterations in the gut microbiome, impaired intestinal barrier function, and immune dysregulation play key roles in the development of these diseases (Guan, 2019; Mehandru and Colombel, 2021).

Because of their multifaceted roles in gut homeostasis, which include maintenance of the stem cell niche (Rosa et al., 2021a), providing structural support (Pieri et al., 2008), regulation of gut motility (Banciu et al., 2022), and modulation of the immune system (Zhang and Tian, 2023), telocytes are thought to be involved in the development of IBD. However, our understanding of the role of telocytes in IBD is still in its nascent stages. In this review, we will describe what is known about intestinal telocytes, their functions in the maintenance of gut homeostasis, their role in the development of IBD, and their potential as novel therapeutic targets.

## 2 Telocyte structure and location in the GI tract

The telocyte nucleus has been described as oval-shaped and contains moderately dense chromatin (Faussone Pellegrini and Popescu, 2011). The surrounding cytoplasm is scarce in volume and contains few rough/smooth endoplasmic reticulum cisternae, a small Golgi apparatus, and some mitochondria. A defining feature of telocytes is the presence of one to five long, thin, monolithic prolongations known as telopodes, measuring tens to hundreds of micrometers in length. The telopodes are comprised of podomeres, thin segments measuring less than 200 nm in diameter; podoms, which are dilated regions containing endoplasmic reticulum and mitochondria; and caveolae (Shoshkes-Carmel, 2024). The number of telopodes dictates the shape of the cell body; one results in a piriform shape, two a spindle, three a triangular, and four a stellate (Popescu and Faussone-Pellegrini, 2010b).

Intestinal telocytes display different morphology and distribution depending on their location in the intestinal wall, which comprises of mucosa, submucosa, muscularis propria, subserosa, and serosa. In the mucosal *lamina propria* of normal ilea, telocytes were initially reported to surround glandular crypts (Milia et al., 2013). However, more recent reports suggest that telocytes are present throughout the ileal mucosa and interface with several cell types including epithelial cells, immune cells, smooth muscle cells, fibroblasts, nerve bundles, and blood vessel (Shoshkes-Carmel, 2024). By contrast recent work by Cadinu et al. (2024) suggested that under colonic homeostasis mucosal telocyte located mostly in subepithelial space at the top of the crypts. In the *muscularis mucosae*, telocytes are numerous, oriented parallel to each other, and display two long, thin telopodes, giving them a spindle shape (Milia et al., 2013).

In the submucosal layer, telocytes display two or three telopodes and roundish cell bodies, are numerous, and form an almost continuous layer bordering the circular *muscularis propria*. They are concentrated around blood vessels, closely encircling the adventitial layer, and are found among vascular smooth muscle cells. Networks of telocytes wrap around lymphatic vessels (Milia et al., 2013). The *muscularis propria* consists of two muscle layers, inner circular and outer longitudinal; between them lies the myenteric plexus, a group of ganglia that innervates the GI tract and facilitates peristaltic movement (Shahrestani, 2024). Within this layer, telocytes have been found to be distributed around and within the circular and longitudinal muscle layers and in the myenteric plexus. They display triangular or stellate cell bodies with three to four telopodes running around muscle fibers; some are embedded in connective tissue between and running parallel to muscle bundles. In the myenteric plexus, telocytes, along with interstitial cells of Cajal (ICC), form a network which encircles the ganglia; the telopodes facilitate intermingling between neurons and glia (Milia et al., 2013).

In the subserosa, like in the muscularis propria, telocytes run in connective tissue. They were also found to be wrapped around blood vessels and neurons and located among adipocytes (Milia et al., 2013).

## 3 Cellular markers and animal models for studying telocytes

Much of the challenge in telocyte biology is the identification of appropriate telocyte markers (Table 1). Several groups have considered FoxL1, PDGFRα, CD34, and Gli1 as intestinal telocyte markers (Shoshkes-Carmel, 2024). Additionally, CD81 has been used in conjunction with the previously described markers to differentiate telocytes from other mesenchymal populations (McCarthy et al., 2020; Kraiczy et al., 2023; Pærregaard et al., 2023; Cadinu et al., 2024). However, on their own, these markers lack specificity. These markers are expressed by other types of cells, including stem cells and other mesenchymal cells; this complicates the definitive identification and functional characterization of telocytes (Tippett et al., 2013; Levy, 2014; Kramann et al., 2015; Miyashita et al., 2020; Muhl et al., 2020; Diaz-Castro et al., 2021; Zurzu et al., 2021).

**TABLE 1:** Characteristics of gastrointestinal telocyte markers.

Marker	Species in which telocytes were studied	Structure/Function	Other cell types that express marker
CD34	Human (Pieri et al., 2008; Milia et al., 2013; Manetti et al., 2015), Mouse (Stzepourginski et al., 2017)	Transmembrane phosphoglycoprotein involved in cellular proliferation, lymphocyte adhesion, and cellular development (Radu et al., 2023)	Hematopoietic stem cells, mesenchymal stem cells, vascular and lymphatic endothelial cells (Sidney et al., 2014; Zurzu et al., 2021)
FoxL1	Possibly human (as ‘stromal-2’ group in scRNA-seq studies by Kinchen et al.) (Kinchen et al., 2018; Kaestner, 2019), Mouse (Shoshkes-Carmel et al., 2018)	Forkhead helix-box L1 transcription factor; target of hedgehog signaling pathway (Katoh and Katoh, 2009)	Hepatic progenitor cells (Shin et al., 2011), lung fibroblasts (Miyashita et al., 2020)
PDGFRα	Human (Vannucchi et al., 2013), Mouse (McCarthy et al., 2020; Kraiczy et al., 2023)	Platelet-derived growth factor receptor alpha; receptor tyrosine kinase involved in embryonic development, cell proliferation, survival and chemotaxis (Andrae et al., 2008)	Mesenchymal stem cells (Farahani and Xaymardan, 2015), fibroblasts (Muhl et al., 2020)
Gli1	Mouse (Degirmenci et al., 2018)	GLI family zinc finger 1, transcriptional effector of hedgehog signaling pathway; involved in stem cell maintenance, fibrosis, development, and cancer pathways (Avery et al., 2021)	Mesenchymal stem cells in various organs (Kramann et al., 2015)
CD81	Mouse (McCarthy et al., 2020; Kraiczy et al., 2023; Cadinu et al., 2024)	Also known as TSPAN28, tetraspanin-28, and TAPA1; surface membrane protein involved in immune response	Macrophages and monocytes (Tippett et al., 2013), lymphoid cells (Levy, 2014), and astrocytes (Diaz-Castro et al., 2021)

Additionally, markers that have been used for telocyte identification have also varied depending on their location in the GI tract. For example, subepithelial intestinal telocytes were identified by combining the markers PDGFRα and FoxL1 (McCarthy et al., 2020; Kraiczy et al., 2023). On the other hand, telocytes in the tongue lamina propria and striated muscle were found to be CD34 and PDGFRα positive (Rosa et al., 2019).

**FIGURE 1:**
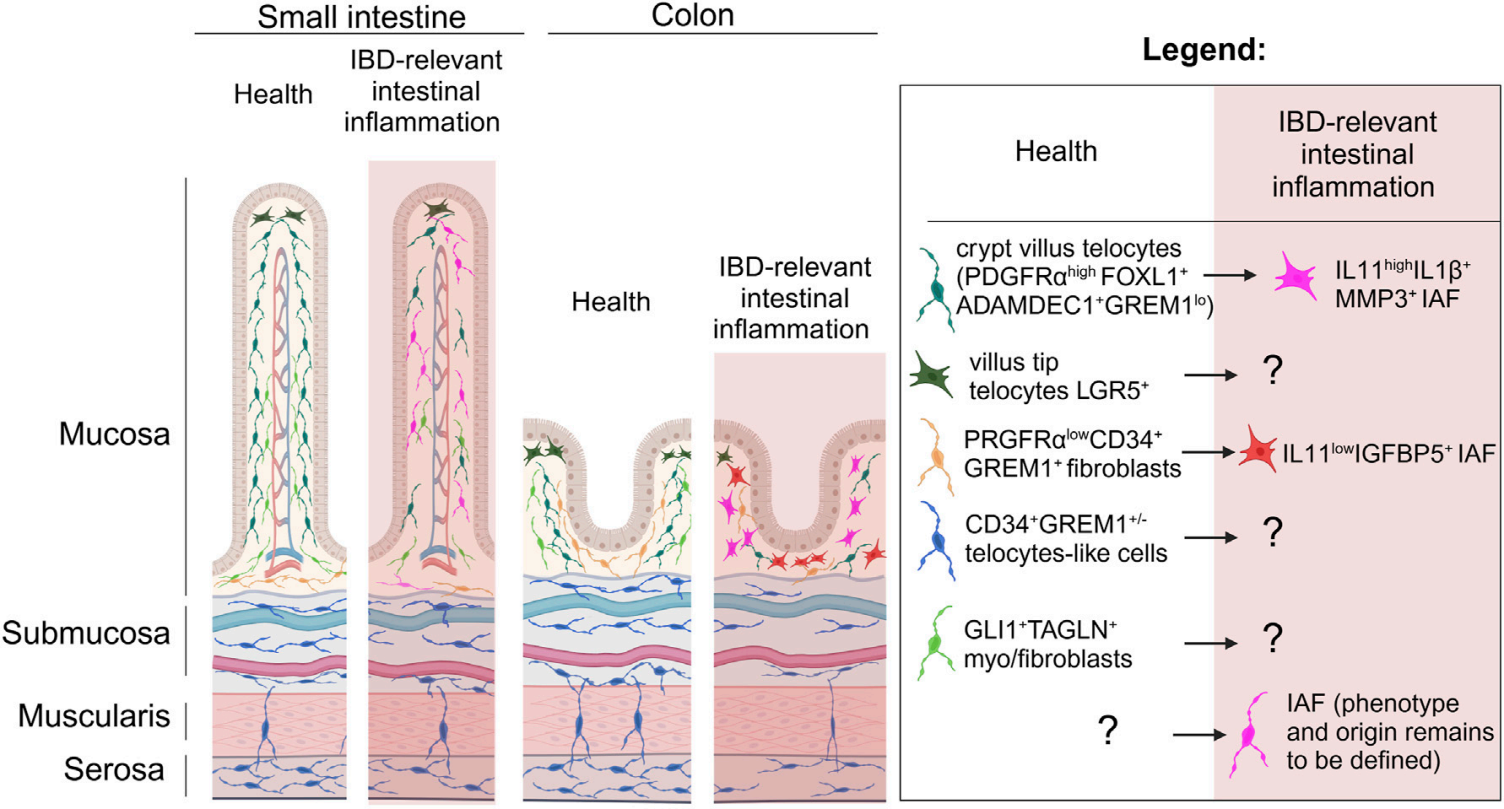
The spatial relationships of telocytes and other mesenchymal cells in the gut under homeostasis and IBD relevant inflammation. Longitudinal representation of gut intestinal villus and colonic crypt show that under homeostasis PDGFRα^high^FOXL1^+^Adamdec1^+^GREM1^low^ telocytes are mostly located in the top of the crypts and likely in the subepithelial space of the villi, and number of these cells is dramatically reduced in IBD relevant inflammation where these telocytes are suggested to be differentiated to IL11^high^IL1β^+^MMP3^+^ IAFs within the crypt. Under homeostasis LGR5^+^ telocytes are found at the top of the villus-crypt axis, while their fate and function in IBD remains to be defined. PDGFRα^low^CD34^+^ GREM1^+^ fibroblasts found at the base of villi and crypts and suggested to differentiate in IL11^low^IGFBP5^+^ IAFs in IBD relevant inflammation. Gli1^+^TAGLN^+^ fibroblasts reported to be distributed within *lamina propria* of the intestinal mucosa and based on transcriptome likely to be myo-/fibroblasts, while CD34^+^ GREM1^+/−^ stromal cells previously described as telocytes are located across submucosa, muscularis propria and serosa in small intestine and colon. IAF, inflammation associated fibroblasts. Created in BioRender. Chulkina, M. (2025) https://BioRender.com/b56u896.

Furthermore, different markers have been used to identify telocytes within the different layers of the intestinal wall (Figure 1). While this has not been systematically shown in the GI tract, in the human bladder, Vannucchi et al. (Vannucchi et al., 2014) identified three different types of telocytes using immunohistochemistry and transmission electron microscopy techniques (TEM). The first subtype was PDGFRα^+^calreticulin^+^CD34^−^c-kit^-^α-SMA^-^ and was found in the more superficial aspect of the suburothelium (Vannucchi et al., 2014). Deeper in the suburothelium, a second telocyte subtype was found with similar markers, except for α-SMA positivity (Vannucchi et al., 2014). These telocytes were identified to be distinct from myofibroblasts based on their TEM characteristics. In the submucosa and detrusor layers, a third type of telocyte was found to be calreticulin^+^ but negative for all the other markers, including PDGFRα (Vannucchi et al., 2014). The variability in marker expression across different tissue types add another layer of complexity in understanding telocyte biology (Ahmed and Hussein, 2023).

Despite the challenges in utilizing the previously described markers in identifying telocytes, some have been successfully used for *in vivo* studies of telocyte function in murine models. The Kaestner lab has developed and utilized several variations of the FoxL1-Cre transgenic mouse to study FoxL1^+^ telocytes (Sackett et al., 2007; Shoshkes-Carmel et al., 2018; Kolev et al., 2021). The Shivdasani lab has used PDGFRα-Cre (Chung et al., 2018) and PDGFRα-GFP (Hamilton et al., 2003) mice to study PDGFRα^+^ telocytes (McCarthy et al., 2020; Kraiczy et al., 2023). The Basler lab has used the Gli1-Cre mouse (Ahn and Joyner, 2004) to study Gli1^+^ telocytes in the gut as well (Degirmenci et al., 2018).

## 4 Telocytes in gut homeostasis and IBD

In general, there is a dearth of studies exploring the role of telocytes in IBD. In this section, we focus on published reports examining telocytes in relation to gut barrier maintenance and repair, and immune system regulation. While our primary emphasis is on studies involving preclinical IBD models and tissues from IBD patients, we also include research on telocyte roles in pathways involved in IBD pathogenesis and in other diseases with similar pathophysiology.

### 4.1 Role of telocyte in the maintenance of the epithelial barrier and stem cell niche

Telocytes have been shown to be important in the maintenance of gut barrier function. In a murine preclinical model of IBD, disruption of BMP signaling in FoxL1^+^ telocytes results in worsened clinical and histologic severity of acute dextran sulfate sodium (DSS) colitis (Reyes Nicolás et al., 2021). These mice exhibited impaired intestinal barrier with abnormal goblet cell maturation and dysregulation of mucin production resulting in increased susceptibility to bacterial mucosal invasion. In another study by the same group, disruption of BMP signaling in FoxL1^+^ telocytes resulted in dysregulated biodynamics of the extracellular matrix and disruption of the colonic collagen network, a process relevant to IBD-associated fibrosis (Pomerleau et al., 2023). In both acute and chronic DSS colitis murine models, CD34^+^GP38^+^α-SMA^-^ pericryptal telocytes have been shown to facilitate intestinal healing and repair through expression of GREM1 (Stzepourginski et al., 2017). There is some conflict, however, whether these cells are to be regarded as telocytes or trophocytes (McCarthy et al., 2020) or if there is the existence of plasticity between these subpopulations of mesenchymal cells.

Telocytes have been demonstrated to play critical roles in supporting the intestinal stem cell niche (Kolev and Kaestner, 2023). Depletion of FoxL1^+^ telocytes in adult mice resulted in grossly shorter small and large intestines (Aoki et al., 2016). These mice exhibited significantly shortened villi and shallower colonic crypts. Depleting FoxL1^+^ telocytes also resulted in abrupt cessation of proliferation of both epithelial stem- and trans-amplifying progenitor populations. Subepithelial FoxL1^+^ telocytes have been shown to be important sources of Wnt ligand (Shoshkes-Carmel et al., 2018). Blocking Wnt secretion in these cells by conditionally knocking out the porcupine gene (PORCN) in FoxL1^+^ telocytes resulted in rapid cessation of Wnt signaling in intestinal crypts. Similarly, selective disruption of Wnt signaling in Gli1^+^ cells, achieved through conditional knockout of the Wntless gene in these cells, resulted in compromised colonic barrier due to impaired stem cell renewal (Degirmenci et al., 2018). Although Gli1 is recognized as a marker for various stromal cell populations (Schneider et al., 2017), it has also been also used as a marker for telocytes (Shoshkes-Carmel, 2024).

An early report by Díaz-Flores et al. (2015) suggested that human intestinal tissue telocytes identified by CD34 are mostly located in the *muscularis mucosae*, submucosa, muscular propria, and serosa, but are absent in the mucosal lamina propria. This is in stark contrast with a previous report by Milia et al. (2013) which reported the presence of CD34^+^ telocytes in the ileal *lamina propria*. Indeed, recent studies using a combination of PDGFRα and FoxL1 as markers of telocytes demonstrated the presence of these cells along the entire crypt-villous axis in the enteric mucosa (McCarthy et al., 2020; Kraiczy et al., 2023). These telocyte populations have been shown to be heterogenous and suggested to provide different signaling factors depending on their location (McCarthy et al., 2020; Kraiczy et al., 2023; Bahar Halpern et al., 2020). FoxL1-expressing PDGFRα^hi^ telocytes have been found to be concentrated at the crypt-villous junction and are an important source of BMP, which allows for the Wnt-BMP gradient to form in the crypt-villous axis (McCarthy et al., 2020; Kraiczy et al., 2023). In a recent study, Lgr5^+^ villous tip telocytes have been shown to be an important signaling source of noncanonical Wnt ligand, and their ablation resulted in long-term altered expression of villous tip genes such as adenosine deaminase (ADA), EGFR, and Fos (Bahar Halpern et al., 2020). These villous tip telocytes are important in regulating epithelial differentiation and endothelial polarization.

In addition to being important sources of BMP/Wnt signaling ligands, telocytes provide structural support to the organs in which they are present. In the gut, CD34^+^ telocytes, previously referred as interstitial Cajal-like cells, were found in the muscularis propria, extending from the stomach to the colon (Pieri et al., 2008). The cells form a complex network by connecting smooth muscles, ICC, nerve bundles, and stem cell niches. These are hypothesized to maintain normal peristalsis in the GI tract and prevent structural deformation (Wei et al., 2022). In the developing mouse heart, telocytes guide myocardial precursors to form the correct three-dimensional tissue pattern (Bani et al., 2010). In human bladders obtained from cystectomy, Vannucchi et al. (2014) found telocytes that were suspected to form a three-dimensional scaffold that allows for normal bladder wall distention and relaxation without resulting in bladder deformation.

### 4.2 Role of telocytes in immune regulation and IBD-relevant intestinal inflammation

Thus far, the role of telocytes in modulating intestinal inflammation is unclear. Telocytes are suggested to function as immune modulators (Zhang and Tian, 2023), and their loss or change likely contributes to inflammation in the gut (Milia et al., 2013; Díaz-Flores et al., 2015). Using acetic acid-induced colitis in rats as an IBD-relevant model, Arafat et al. (2021) showed that nanocurcumin treatment results in an increased number of intestinal CD34^+^ telocytes, identified *in situ* based on positivity for CD34 and mesenchymal marker vimentin, and significantly attenuated the severity of this experimental model of colitis. Despite this, the specific mechanisms involved in the decrease of population of these cells and how this contributes to inflammation remain unknown.

In a recent spatial transcriptomics-based study, Cadinu et al. (2024) mapped the biogeography and trajectories of fibroblasts throughout the course of acute DSS-induced murine colitis. Using state of art multiplex error-robust fluorescence *in situ* hybridization (MERFISH), the authors examined the cellular remodeling that occurs in the colon during different stages (homeostasis, early acute inflammation, peak acute inflammation, and recovery phase) of a DSS-induced murine colitis model (Cadinu et al., 2024). In this work, four different major fibroblast subsets were identified using the markers CD34, PDGFRα, Gli1 that were previously ascribed to telocytes. Notably, during homeostasis, two distinct CD34^+^ fibroblast populations, fibro 6 and fibro 7, were identified in the colonic mucosa and submucosa, respectively. These subsets were dramatically reduced during acute inflammation. The CD34^+^ fibro 6 subset was mostly seen at the base of the colonic crypt and found to express Wnt2b, Col15α1, and GREM1 (Cadinu et al., 2024). The CD34^+^ fibro 7 subset was enriched in the submucosa and expressed Pi16, Ackr4, C3, and GREM1. Both the fibro 6 and fibro 7 subsets expressed low levels of PDGFRα (PDGFRα^low^) and were linked to the CD81^+^PDGRFα^low^ GREM1-producing cells referred to as trophocytes (McCarthy et al., 2020).

Another telocyte marker, Gli1, was assigned to fibro 13, which was distributed through the mucosal lamina propria (Cadinu et al., 2024). Similar to the CD34^+^ fibroblast subsets, the Gli1^+^ fibro 13 population is also largely present during homeostasis and their population tends to decrease during inflammation. This fibroblast population expressed Tagln, Gli1, and other integrins and, as such, were thought to be myofibroblasts (Cadinu et al., 2024).

Under homeostasis, the fibro 2 subset, identified as PDGFRα^high^, was found at the top of colonic crypt. These cells expressed Adamdec1, Wnt5a, Bmp2, Bmp5, Bmp7, assigning them to the previously termed “crypt-top fibroblasts” or “telocytes” (Cadinu et al., 2024). Previously, this subset was also linked to PDGFRα^high^ Adamdec1^high^ fibroblasts that also express the telocyte-linked marker FoxL1 (Shoshkes-Carmel et al., 2018; Jasso et al., 2022).

The four fibroblast subsets characterized by Cadinu *et al.* that express markers thought to be associated with telocytes (i.e., CD34, Gli1, and PDGFRα) are present at the early stage of acute colitis; these subsets were dramatically reduced at the peak of acute inflammation in DSS colitis (Cadinu et al., 2024). Based on its geographic distribution and transcriptomic changes, Cadinu *et al.* suggested that PDGFRα^low^ CD34^+^GREM1^+^ fibro 6 give rise to a subset of crypt-base enriched IL-11^low^ inflammation-associated fibroblasts (IAFs) marked by expression of Igfbp5, GREM1, Col18α1, and Mmp2 (Cadinu et al., 2024). This population of IAFs start to appear at the initiation of inflammation and become most abundant at the peak inflammation phase of acute colitis. By contrast, the Adamdec1^+^PDGFRα^high^ fibro 2 population appears to give rise to a population of IAFs with high levels of IL-11, IL-1β, IL-1rl1, Mmp3, Mmp10, Mmp13, and Plau (Cadinu et al., 2024). This IL-11^high^ IAFs derived from fibro 2 only appears during the peak inflammatory stage of acute DSS colitis and is largely undetectable at the beginning of inflammation. Interestingly, in the context of chronic colonic inflammation, Jasso et al. (2022) previously showed (scRNAseq analysis) that in chronic DSS colitis, several fibroblast subsets expressed the IL-6 family cytokine IL-11 and the receptor subunits IL-11ra and IL-6th (Jasso et al., 2022). As such, the authors suggested a putative role for the IL-6 cytokine family in the intercommunication between different fibroblast subsets, as well as with endothelial cells (Jasso et al., 2022). The fate of the CD34^+^ fibro seven and Gli1^+^ fibro 13 subsets are less clear in this murine model of colitis (Cadinu et al., 2024).

While Cadinu et al. (2024) work highlights the transcriptomic changes in telocytes during inflammatory stress, little is known about how these changes in gene expression affect intestinal telocyte morphology. Interestingly, recent work by Emeish et al. (2023) examining intraepithelial and stromal telocytes in common carps during salinity-induced stress demonstrated that these telocytes undergo cellular hypertrophy with increased secretory vesicles and changes in mitochondrial shape. In addition, CD34^+^ adipose tissue telocytes in elastofibrolipoma, a benign fibrotic tumor, display a bead or globular structure when located between mature adipocytes (Díaz-Flores et al., 2020). By contrast, telocytes in elastofibrolipoma appear bulky and have a variable number of telopodes (Díaz-Flores et al., 2020). Thus, it is likely that the transcriptomic changes observed in intestinal telocytes during IBD-relevant inflammatory stress would be accompanied by morphological changes as well.

In addition to transcriptomic studies, various groups have also explored the cellular and molecular mechanisms by which telocytes regulate the immune system. Using immunohistochemistry and TEM techniques, Ji et al. (2021) showed that PDGFRα^+^ cells which the group identified as telocytes, directly interact with macrophages in the intestinal *muscularis propria* of both mouse- and human-derived tissue. The importance of this interaction has not been explored in the context of IBD; however, in a mouse model of periodontitis, an inflammatory disease characterized by the pathologic increase in the expression of TNF-α, IFN-γ, and IL-6 similar to IBD, telocytes have been shown to promote a shift in macrophage polarization from the pro-inflammatory M1 state to a tissue-remodeling M1/M2 state via the HGF/c-Met signaling pathway (Zhao et al., 2022). In this murine model of periodontitis, telocytes were defined as CD34^+^CD31^−^ cells that express hepatocyte growth factor (HGF), which was reported to bind to c-Met receptors on periodontal macrophages, resulting in decreased expression of iNOS and increased expression of Arginase one in macrophages, consistent with a transition from an M1-to an M2-like state (Zhao et al., 2022). Importantly, in IBD, aberrant intestinal macrophage polarization is thought to contribute to disease development and resistance to therapy (Du et al., 2021).

### 4.3 Role of telocyte in the intestinal repair: Potential role in IBD-associated fibrosis

There is burgeoning data showing that loss of telocytes or impairment in telocyte function contributes to the development of fibrosis. Decrease or complete loss of CD34^+^ stromal cells identified as telocytes has been reported in various diseases associated with fibrotic complications, such as systemic sclerosis (Manetti et al., 2013), heart failure (Richter and Kostin, 2015), liver fibrosis (Fu et al., 2015), gynecologic diseases (Varga et al., 2018; Xu et al., 2023), and IBD.

While the nature of the decrease in CD34^+^ telocyte number and how this contributes to the development of fibrosis remain unclear, these cells have been suggested to counteract fibrosis by HGF-mediated inhibition of the TGF-β1/Smad signaling pathway in the rat model of renal fibrosis (Zheng et al., 2018) and by enhancing mesenchymal to epithelial transition during the process of decidualization in endometrium (Zhang F.-L. et al., 2020).

In IBD, fibrosis is believed to be secondary to chronic inflammation and is characterized by the excessive deposition of the extracellular matrix (D’Alessio et al., 2022). In CD, fibrosis can occur throughout all layers of the intestinal wall (D’Alessio et al., 2022); meanwhile, in UC, fibrosis is proposed to be limited to the colonic mucosa, but this requires better characterization (Gordon et al., 2018).

In disease-affected areas of ileal CD, Milia et al. (2013) reported that the number of CD34^+^PDGFRα^+^ telocytes inversely correlated with the severity of CD-associated fibrosis. In this study, full thickness ileal specimens obtained from patients with and without CD were compared *in situ*. Telocytes, identified by CD34 and PDGFRα, were markedly decreased in areas of intestinal fibrosis. Further, at the myenteric plexus, the loss of telocytes paralleled the loss of ICC. Similar findings were demonstrated by Arslan *et al.* who used CD34 as a surrogate marker of telocytes and quantified *in situ* CD34 positive staining in surgically-resected ileal tissue from patients with stricturing and inflammatory CD (Arslan et al., 2022). In areas of stricturing CD, fewer CD34^+^ telocytes were observed compared to unaffected areas of the small intestine. Like CD, a decreasing number of CD34^+^ telocytes also correlated with the presence of fibrotic changes in UC (Manetti et al., 2015). In UC with minimal fibrotic changes, the number of telocytes are decreased in both the muscularis mucosa and submucosa. Moreover, telocytes were rarely found or even completely absent in the myenteric plexus of patients with advanced fibrotic UC. Despite these recent publications describing telocytes in IBD, the cause of the decreased number of telocytes, their fate, and their exact functional contribution to fibrosis in IBD is not known. Although further experimental validation (through lineage tracing experiments, for example,) is needed, Diaz-Flores et al. (2015) suggest that telocytes in inflamed appendicitis, diverticulitis, and CD tissue lose CD34 positivity and differentiate into α-SMA^+^ myofibroblasts. In addition, loss of telocytes in patients with IBD has been suspected to be the pathologic mechanism responsible for gut dysmotility observed in this disease (Banciu et al., 2022).

While most of the work on understanding the function of telocytes as it relates to fibrosis remains descriptive, a recent study using a chronic DSS murine colitis model suggests the importance of IL-11-producing fibroblasts in IBD-relevant fibrosis (Jasso et al., 2022). Interestingly, in an acute DSS colitis model utilized by the same group, Adamdec1 was found to be critical for colonic repair (Jasso et al., 2022). Adamdec1 was also reported to be highly expressed by PDGFRα^high^ telocytes (Cadinu et al., 2024), suggesting that these cells may be important in profibrotic remodeling in IBD.

## 5 Telocytes as novel therapeutics in immune-mediated and fibrotic diseases

Over the years, it has become increasingly clear that the mesenchymal cell population recently identified as telocytes have unique roles in tissue repair, immune regulation, and maintenance of structural integrity. These functions position them as promising candidates for developing treatment approaches aimed at mitigating fibrosis and modulating the immune response. Although there have been no studies exploring therapeutic roles for telocytes specifically in IBD, telocyte transplantation has been investigated in several other preclinical models of inflammatory and fibrotic diseases and found to alleviate their target disease phenotypes.

In a rat model of myocardial infarction, intramyocardial injection of cardiac CD34^+^c-Kit^+^ telocytes resulted in decreased infarction size and improved myocardial function (Zhao et al., 2013). Using a unilateral ureteral obstruction-induced renal fibrosis in a rat model, Zheng et al. (2018) demonstrated that tail-vein administration of cultured CD34^+^c-Kit^+^ telocytes resulted in disease attenuation as evidenced by reduced renal collagen accumulation and decreased expression of profibrotic genes. In a mouse model of lipopolysaccharide (LPS)-induced skin wound formation, intradermal injection of CD34^+^c-Kit^+^ PDGFRα^+^ telocytes resulted in decreased wound healing delay and reduced inflammatory responses (Wang et al., 2020). Co-transplantation of CD34^+^ telocytes and mesenchymal stem cells have also been demonstrated to improve lung injury scores in a mouse model of LPS-induced lung injury. This combined treatment led to a statistically significant decrease in lung injury when compared to treatment with either cellular therapies alone (Zhang D. et al., 2020). Apart from telocyte transplantation, telocyte-derived products have also been shown to alleviate preclinical models of disease. In a mouse model of LPS-induced endometrial fibrosis, intrauterine injection of telocyte-derived exosomes decreased uterine fibrosis and enhanced mesenchymal-to-epithelial transition (Chen et al., 2023).

## 6 Discussion

The emerging understanding of telocytes and their roles in tissue homeostasis, particularly in the gastrointestinal tract, highlights their potential role in the pathogenesis of IBD. However, the functional significance of telocytes and if the functions of these cells vary based on their location in the intestinal wall (i.e., lamina propria *versus* muscularis propria) and along the length of the gut (i.e., in stomach telocytes *versus* colonic telocytes) remains to be defined. The recent work by Cadinu et al. (2024) taken together with the previous finding by McCarthy et al. (2020) suggest that there may be some plasticity between subpopulations of mesenchymal cells, including telocytes, trophocytes and IAFs during IBD-associated inflammation. To this end, single-cell multi-omic mapping of telocytes in the gut as well as lineage tracing experiments need to be done in the future.

The challenge with the aforementioned approach is related to the lack of uniformity and consensus in the markers used to identify telocytes. While FoxL1, PDGFRα, CD34, and Gli1 have been used to identify telocyte in the gut (Shoshkes-Carmel, 2024); these markers were also found to be expressed by other types of cells including stem cells and other mesenchymal cells, which complicates the definitive identification and functional characterization of telocytes (Table 1) (Tippett et al., 2013; Levy, 2014; Kramann et al., 2015; Miyashita et al., 2020; Muhl et al., 2020; Diaz-Castro et al., 2021; Zurzu et al., 2021). As such, it remains debatable if telocytes are a distinct differentiated subset within mesenchymal cells or another transcriptomic stage of fibroblasts to the changes in the intestinal microenvironmental cues. Indeed, Cadinu et al. (2024) showed that CD34, PDGFRα, and Gli1 likely define different subsets of fibroblasts.

The GREM1-producing Gli^+^GP38^+^ pericryptal telocytes identified by Degirmenci et al. (2018) are likely the same GREM1-producing PDGFRα^low^ trophocytes identified by McCarthy et al. (2020). These trophocytes were also subsequently described by Jasso et al. (2022), Pærregaard et al. (2023), and Cadinu et al. (2024) among others. These PDGFRα^low^ trophocytes are differentiated from another PDGFRα^low^ fibroblast subset based on their CD81 expression. Both of these PDGFRα^low^ subsets are also CD34^+^. Whether these CD34^+^PDGRα^low^ fibroblast subsets, such the CD81^+^ trophocytes and CD81^−^ fibroblasts, should be classified as telocyte subtypes is currently uncertain. Much of the early studies on telocytes focus on the use of the CD34 marker (Faussone Pellegrini and Popescu, 2011; Milia et al., 2013; Manetti et al., 2015; Ibba-Manneschi et al., 2016). In addition, in other organs, such as skin and lungs, CD34 positivity is still often associated with telocytes (Rusu et al., 2020; Díaz-Flores et al., 2021).

More recently, PDGFRα^high^FoxL1^+^ fibroblasts present at crypt-tops and villous tips are more likely to be referred to as telocytes in the intestinal stroma literature (Shoshkes-Carmel et al., 2018; Bahar Halpern et al., 2020; McCarthy et al., 2020; Kraiczy et al., 2023; Cadinu et al., 2024). Interestingly, recent study by Pærregaard et al. (2023) suggested that this population of cells comprise functionally distinct subpopulations of cells originated from an Fgfr2-expressing stromal cells. These studies altogether suggest that some of the populations previously identified as telocytes are not uniform and may comprise several different fibroblast subpopulations. These fibroblast subpopulations also appear to be highly plastic instead of being terminally differentiated and may acquire or lose functions depending on microenvironmental cues that are present. The potential roles of these different stromal cell populations and their fate/plasticity during pathogenesis of IBD remain to be defined.

Over the last decade, considerable progress has been made in understanding the role of stromal cells often referred to as telocytes in gut barrier function. Disruption of Wnt and BMP signaling pathways in FoxL1^+^ telocytes has been shown to impair these functions, leading to compromised barrier integrity and abnormal tissue dynamics (Reyes Nicolás et al., 2021; Pomerleau et al., 2023). However, it remains to be seen how Wnt/BMP signaling in telocytes protect against “leaky” gut (Camilleri, 2019) and how these disruptions in telocyte function can potentially exacerbate IBD symptoms by allowing increased microbial translocation and perpetuating chronic inflammation. The correlation between the loss of telocytes and the presence of fibrosis in IBD and other diseases suggests that these cells are likely to play a protective role against the development of fibrosis (Wei et al., 2022). The inverse correlation between the number of telocytes and fibrosis severity, as reported in studies involving CD and UC, suggests that telocyte depletion or trans-differentiation into other subsets of mesenchymal cells, likely inflammation-associated fibroblasts (IAF), may contribute to or exacerbate the inflammatory and fibrotic processes observed in these diseases (Milia et al., 2013; Manetti et al., 2015).

The field of telocyte biology is only in its early stages when it comes to understanding how telocytes interact with other cell types that are known to be key players in IBD-associated chronic inflammation and fibrosis, such as epithelial cells, fibroblasts, smooth muscle cells, and capillary wall cells. For example, through telopodes, telocytes are suggested to form direct cell-cell contact with various epithelial cells, endothelial cells, and fibroblasts (Song et al., 2022; Fu et al., 2023). However, the overall role and mechanisms by which telocytes regulate the responses of other cells in chronic inflammation and fibrosis is far from understood and, indeed, some findings remain contradictory. In a different study, skin telocytes were shown to inhibit the release of inflammatory factors and promote migration of epithelial cells in *in vitro* and *in vivo* models of skin injury (Wang et al., 2020). However, in another study of dermal fibrosis, telocytes were also suggested to be a source of α-SMA^+^ myofibroblasts, a recognized contributor to fibrosis (Rosa et al., 2021b). In models of pulmonary fibrosis, telocytes are shown to suppress excessive activation of fibroblasts and endothelial cells by regulating their proliferation, differentiation, and matrix production, potentially reversing excessive collagen deposition (Zheng et al., 2024). Similar findings were noted in liver fibrosis (Wei et al., 2022). Indeed, in models of myocardial infarction and renal fibrosis, there is some evidence that telocyte transplantation contributes to the reduction of extracellular matrix deposition and enhances recovery of organ function (Wei et al., 2022). The role of telocytes in regulating capillary wall cells, including endothelial cells, during IBD also requires further investigation. A recent study suggests that telocytes may facilitate angiogenesis during tissue development and repair through expression of VEGF which promotes endothelial proliferation and migration (Soliman, 2021). From a smooth muscle interaction standpoint, intestinal telocytes have been suggested to control smooth muscle contraction in the GI tract (Shoshkes-Carmel, 2024). Nonetheless, the mechanisms by which telocytes regulate activity of other major cellular players in chronic inflammation and fibrotic stricture formation in IBD remain to be elucidated.

The role of telocytes in immune regulation, particularly their interaction with macrophages, suggests a broader immunomodulatory function that could be leveraged for therapeutic purposes. While Ji et al. (2021) have previously shown that PDGFRα^+^ telocytes form cell-to-cell contacts with macrophages in the intestinal muscle layers, the nature and mechanisms of these interactions between macrophages and telocytes in other layers of the intestinal wall during homeostasis and IBD remain to be defined. The ability of telocytes to influence macrophage polarization via the HGF-Met pathway from an inflammatory (M1) state toward a tissue-remodeling phenotype (M1/M2) in a preclinical model of periodontitis (Zhao et al., 2022) hints at the potential of telocyte-macrophage interactions in IBD and its associated fibrosis. Future studies will need to elucidate the impact of telocyte-macrophage interactions on IBD-associated chronic inflammation and fibrosis.

Further, because telocytes are suspected to transdifferentiate into IAF, it is unclear if telocyte transplantation will add more fuel to fire as the transplanted telocytes potentially become more sources of IAF precursors. Alternatively, these transplanted telocytes could potentially revert the transcriptomic programming of IAFs back into their resting state and halt IBD-associated inflammatory and fibrotic processes. Our understanding of the immunoregulatory function of telocytes in the context of gut homeostasis and IBD is still in its infancy. Further work is needed to elucidate the mechanisms by which telocytes influence immune responses, inflammation, and IBD-associated fibrosis. This will involve not only the identification of specific markers and signaling pathways but also the development of models to study telocyte function *in vitro* and *in vivo*.

In conclusion, our knowledge of telocyte fate, plasticity, and function during gut homeostasis and IBD-relevant inflammatory stress remains in its nascent stages. Their multifaceted roles in maintaining the gut barrier and regulating innate immune responses and potentially protective role against fibrosis make them an attractive novel therapeutic target for IBD. Approaches such as enhancement/restoration of telocyte function or administration of telocyte-derived products should be considered as new strategies to manage fibrosis and inflammation in IBD.
